# Hypertension among human immunodeficiency virus infected patients on treatment at Parirenyatwa Hospital: A descriptive study

**DOI:** 10.4102/phcfm.v11i1.1974

**Published:** 2019-08-19

**Authors:** Rumbidzai Chireshe, Keshena Naidoo, Rudo Nyamakura

**Affiliations:** 1 Discipline of Nursing and Public Health, University of KwaZulu-Natal, Durban, South Africa; 2 School of Nursing, Public Health and Family Medicine, University of KwaZulu-Natal, Durban, South Africa; 3 Department of Nursing Science, College of Health Sciences, University of Zimbabwe, Harare, Zimbabwe

**Keywords:** hypertension prevalence, HIV-positive, highly active antiretroviral therapy, sub-Saharan Africa, protease inhibitors, antiretroviral therapy, non-communicable diseases

## Abstract

**Background:**

Since the roll-out of antiretroviral therapy (ART) in sub-Saharan Africa (sSA) in the early 2000s, the life expectancy of people infected with the human immunodeficiency virus (HIV) has increased. However, the gains made in reducing mortality from HIV-related complications have been mitigated by the emergence of age-related chronic non-communicable diseases (NCDs), such as hypertension. Protease inhibitors (PIs), and prolonged exposure to highly active ART (HAART) have been implicated in the development of hypertension in HIV-positive people.

**Aim:**

To investigate the prevalence of hypertension and its associated risk factors among HIV-positive patients receiving ART.

**Setting:**

The study was carried out at an urban-based clinic that provides HAART and primary care to HIV-positive people in Harare, Zimbabwe.

**Methods:**

A descriptive, cross-sectional study was conducted among non-pregnant adults on HAART attending the clinic between July and August 2018.

**Results:**

We studied 600 HIV-positive adult patients, of which 56% were women. The prevalence rate of hypertension was 29.9%. Of the participants in the hypertensive group, 11.2% were not previously diagnosed or on treatment. Factors associated with hypertension were advanced age, use of HAART for longer than 10 years, being overweight, a family history of hypertension and smoking. There was a 68.8% prevalence of body mass index greater than 25 kg/m^2^ among all participants.

**Conclusion:**

High hypertension prevalence was recorded. Hypertension was not associated with gender or use of PI regimens but being overweight was highly prevalent. Greater vigilance and integration of resources is required in the overall treatment and monitoring of HIV-positive patients for co-morbidities.

## Introduction

According to statistics from the United Nations, there were over 36.9 million people worldwide in 2017 who were known to be human immunodeficiency virus (HIV)-positive.^1^ Sub-Saharan Africa (sSA) is the most affected, with an estimated two-thirds of all HIV-positive people living in this region.^2^ The availability and use of highly active antiretroviral therapy (HAART) in sSA is expanding rapidly, with 21.7 million patients estimated to be on treatment by the end of 2017.^1^ Since the roll-out of ART almost two decades ago, the mortality rate from HIV-related complications and AIDS has decreased, resulting in improved survival of people living with HIV.^3,4,5^ However, the quality of these extended years is unclear.^6^ Zanetti et al. suggested that HIV-positive patients on HAART may be at increased risk for cardiovascular morbidity.^7^ This could be because of chronic inflammation caused by the HIV, the metabolic effects of HAART or the result of other risk factors in HIV-positive people.^3^ Increased life expectancy is also accompanied by diseases associated with ageing such as hypertension. Hypertension and other chronic non-communicable diseases (NCDs) are an increasing public health concern in sSA.^8^ In a research performed in 2017, it was observed that the prevalence rate of participants who reported themselves as hypertensive ranged from 14.1% in Ghana to 40.8% in Russia, whereas the prevalence rate of measured hypertension ranged from 38.8% in India to 73.8% in South Africa; this may be because of increased life expectancy in sSA, lack of physical activities, smoking, poor diet and obesity.^8^ As yet, there is little data on the prevalence of chronic NCD co-morbidities in the HIV-positive population.

A meta-analysis study published in 2015 showed that the burden of hypertension in sSA has been growing over the years. The average prevalence of hypertension in sSA was estimated at 22.9%.^9^ The prevalence rate of hypertension in Zimbabwe was estimated to be 25% in the general population in 2015.^10^ Factors associated with hypertension were age, smoking and obesity.^10^

Few studies have investigated the prevalence of hypertension or associated factors in HIV-infected people on HAART in sSA. A meta-analysis published in 2017 estimated that 25.2% of HIV-positive people worldwide are hypertensive. However, only ten of the studies were from Africa, and none from Zimbabwe.^11^ Risk factors for hypertension were antiretroviral therapy and older age. Prior to the availability of HAART, hypertension in HIV-positive people was seen as secondary to HIV-related complications such as HIV-associated nephropathy (HIVAN) and vasculopathy.^12,13,14^ Other conventional risk factors for hypertension such as obesity and advanced age are becoming increasingly prevalent in HIV-positive populations.^10,11,15^

A study in Ethiopia investigated the prevalence and risk of metabolic syndrome in HIV-positive people on HAART in 2010. This study found out that those who were taking HAART for a longer duration had higher chances of developing metabolic complications.^16^ Another study that was carried out in Kenya noted that there is a high prevalence of obesity and hypertension among HIV-positive people. This high prevalence varied between women and men.^17^

The main purpose of the research study was to investigate the hypertension prevalence rate and its associated risk factors among HIV-positive patients attending the ART clinic at the Parirenyatwa Central Hospital, Zimbabwe.

## Methods

We conducted a descriptive, cross-sectional study among HIV-positive adult patients attending the opportunistic infections (OI)-ART Clinic at Parirenyatwa Central Hospital between July and October 2018. This health clinic provides HAART to over 13 000 people living with HIV (PLWH) in Harare, Zimbabwe.^8^

### Sampling strategy

Non-pregnant, HIV-positive patients who were 18 years and above were recruited to participate in the study. Pregnant patients were excluded as hypertension in pregnancy is often a self-limiting condition with a different aetiology to primary hypertension. The ART clinic provides care to approximately 1000 HIV-positive patients per month. Approximately 30% of monthly clinic attendees were sampled during the study period. A sample size of 600, in a proportion of 17:20:13 for those on antiretroviral therapy for ≤ 5 years, between 5 and 10 years and ≥ 10 years, was determined after consultation with the biostatistician to be adequately powered to determine the casual relationship between the length of exposure to HAART and the risk for hypertension.

The total sample size was calculated as shown below.

### Sample size calculation

- Estimate of the expected proportion (*p*) of patients who have developed hypertension = 0.5.
- Desired level of absolute precision (*d*) = 0.5.
- Estimated design effect (DEFF) = 1.5.
  $$\begin{array}{l} n=\frac{1.96^{2}p(1-p)(DEFF)}{d^{2}}\\ n=\frac{1.96^{2}\times .5\times .5(1.5)}{.05^{2}}=576.24. \end{array}$$ [Eqn 1]
- Assuming 4% will decline to participate in the study.

Minimum sample size = 576.24 + 23.44 = 599.68.

### Data collection

A structured interviewer-administered questionnaire and physical examination was used to collect data from the patients. The questionnaire was adapted from the World Health Organization STEPS^1^ instrument. Socio-demographic and medical data were collected from participants, that is, age, gender, weight, height, blood pressure record, history of hypertension in the family and duration of HAART. Patient medical records were used to obtain and confirm reported diagnoses, previous blood pressure readings and hypertension medication. Questionnaires were translated into a vernacular language (Shona) and data were collected by the interviewer.

Education was categorised according to the levels of education, namely, primary school, secondary school, tertiary education and no basic education. We collected employment data in pre-coded categories: employed, self-employed, student, unemployed, housewife or husband and retired. We further collected data on salaries using salary brackets of US $100.00 – $300.00, $301.00 – $500.00, above $500.00 and no salary at all.

We also collected data on physical activities with three categories, namely, yes, no and daily chores. ‘Yes’ was for those who were actively doing exercises, while ‘no’ was for those who did not do physical activities, and finally, ‘daily household chores’ was a category for those who were not actively doing exercise but were doing household chores such as gardening, house cleaning and fetching of water and firewood. Data about behavioural risk factors such as smoking and alcohol consumption were also collected. Data collected for smoking were categorised as ‘yes’ for smokers and ‘no’ for non-smokers. Alcohol consumption had three categories, namely, ‘yes’, ‘no’ and ‘occasional drinking’. ‘Yes’ was for those who drink alcohol every day or week, while ‘no’ was for those who did not drink alcohol and the final category was for occasional drinkers.

The body mass index (BMI) of participants was calculated by dividing the weight in kilograms by height in square metres. Weight was assessed using a digital clinical scale and height was measured using a wall-mounted tape measure. Body mass index was classified as follows: below 18.5 kg/m^2^ as underweight, 18.5 kg/m^2^ – 24.9 kg/m^2^ as normal weight, 25.0 kg/m^2^ – 29.9 kg/m^2^ as overweight and above 30 kg/m^2^ as obese.

Blood pressure was measured three times in all participants using a calibrated automated sphygmomanometer machine after the participant had rested for 10 minutes in a quiet room. The average of the last two blood pressure readings was then used for analyses:

Patients were classified as hypertensive if they fulfilled one of the following criteria:

- documentation of hypertension in patient record
- two or more blood pressure readings *of* > 140/90 at two different visits
- self-reporting of prior diagnosis of hypertension.

### Data analysis

Analyses of raw data were performed with the IBM SPSS Statistics version 25. Descriptive statistics and medians were analysed statistically. Estimated odds ratios (ORs) and 95% confidence intervals that were used to check the associations between hypertension and other potential risk factors were calculated using logistic regression. The associations between hypertension and risk factor variables were calculated using the *p*-values. A *p*-value of less than 0.10 was determined to be significant.

A bivariate logistic regression model was also used to examine the relationship between hypertension and age, gender, family history of hypertension, education level, employment status, salary, alcohol consumption as well as clinical variables of BMI and duration of HAART.

### Study validity

All HIV-positive patients attending the Parirenyatwa Hospital Opportunistic Infection (OI) Clinic during the study period were given an equal opportunity to participate in this study. Study bias was avoided by use of a protocol for the collection, measurement and interpretation of information. A standardised questionnaire adapted from a validated tool was used. All data were collected by the primary investigator following the principles of good clinical practice. Medical data provided by participants were verified by medical records to avoid information bias.

### Ethical considerations

Ethical approval was obtained from the University of KwaZulu-Natal Biomedical Ethics Committee (BE085/18) and from the University of Zimbabwe, Joint Research Ethics Committee (JREC133/18).

Written informed consent was obtained from all study participants. Privacy was accorded during interviews to guarantee confidentiality, and no personal identifiers were included on the questionnaire. All due care was taken to avoid discomfort to the participants.

Linkage of care was provided to participants who were diagnosed with hypertension for the first time during this study, and participants known to be hypertensive but not on treatment. They were referred, with consent, to a clinician for further investigation and management.

## Results

### Profile of participants

We enrolled 600 HIV-positive participants into the study, of which 56% (*n* = 334) were women. As described in Table 1, the mean age was 41.8 years (range 18–74 years). The majority of participants were 40 years and older (57%). There was a high literacy rate with over 80% of participants having completed secondary schooling. Most participants were employed (57.5%). The prevalence of being overweight or obese (i.e. a BMI greater than 25 kg/m^2^) was 68.8%. Participants who were on first-line treatment were 70% (*n* = 421), 29% (*n* = 173) were on second-line treatment and 1% (*n* = 6) were on third-line treatment. There was a high level of literacy with over half of the participants having completed secondary school and a further 28% tertiary education. The proportion of participants who were employed was 42% and over half of the participants had an income of more than $300.00 a month (Table 2).

**TABLE 1:** Descriptive profile of adult human immunodeficiency virus–positive participants associated with hypertension at Parirenyatwa Hospital, OI Clinic, in 2018.

Variable	*N*	Mean	Standard deviation	Minimum	Maximum
Age (years)	600	41.8	12.7	18	74
Age categories					
18–39	259	29.8	6.6	18	39
40–59	280	47.9	4.7	40	58
≥ 60	61	64.4	3.9	60	74
BMI	600	29.3	7.4	-	-
BMI categories					
Underweight (< 18.5 kg/m^2^)	29	16.6	1.3	13.5	18.4
Normal (18.5 kg/m^2^ – 24.9 kg/m^2^)	159	22.3	1.7	18.6	25.0
Overweight (25.0 kg/m^2^ – 29.9 kg/m^2^)	160	27.5	1.4	25	30.0
Obese (> 30 kg/m^2^)	252	36.3	5.0	30.0	54.1
Duration on ART (years)	600	6.5	3.7	1	15

BMI, body mass index; ART, antiretroviral therapy.

**TABLE 2:** Descriptive characteristics and factors associated with hypertension in adult human immunodeficiency virus–positive patients at Parirenyatwa Hospital, OI Clinic, in 2018.

Variable	Frequency	Normotensive		Hypertensive		OR	*Z*	*P*-Value	95% confidence interval	
		*N*	%	*N*	%				From	To
Sample size	600	422	70.3	178	29.7	-	-	-	-	-
Age, years										
18–39	259	183	70.7	76	29.3	Base category	-	-	-	-
40–59	280	197	70.4	83	29.7	2.0	2.57	0.01	1.2	3.4
≥ 60	61	42	68.9	19	31.1	4.9	3.82	0.00	2.2	10.9
Gender										
Male	266	182	68.4	84	31.6	0.9	−0.71	0.48	0.6	1.3
Female	334	242	-	94	28.1	Base category	-	-	-	-
BMI										
Below 18.5	29	21	72.4	8	27.6	Base category	-	-	-	-
18.5–24.9	159	115	72.3	44	27.7	0.4	−1.38	0.17	0.1	1.4
25.0–29.9	160	112	70.0	48	30.0	0.5	−1.08	0.28	0.2	1.7
> 30.0	252	174	69.0	78	31.0	0.9	−0.10	0.92	0.3	2.9
Smoking										
No	586	416	71.0	170	29.0	Base category	-	-	-	-
Yes	14	6	42.9	8	57.1	1.0	0.06	0.95	0.2	3.8
Alcohol consumption										
No	517	364	70.4	153	29.6	Base category	-	-	-	-
Yes	7	5	71.4	2	28.6	11.2	2.85	0.00	2.1	59.4
Occasional drinker	76	53	69.7	23	60.3	0.8	−0.74	0.46	0.4	1.5
Physical activity										
No	71	56	78.9	15	21.1	Base category	-	-	-	-
Yes	51	39	76.5	12	23.5	1.0	−0.06	0.96	0.3	2.7
Daily chores as activities	478	327	68.4	151	31.6	0.9	−0.21	0.83	0.5	1.8
Family history of HPT										
No	157	107	68.2	50	31.9	Base category	-	-	-	-
Yes	146	105	71.9	41	28.1	2.0	1.98	0.05	1.0	4.1
Not sure	297	210	70.7	87	29.3	3.0	3.45	0.00	1.6	5.5
Duration on HAART	6.5 (4–10)	6	3–10 (49.3%)	7	5–9 (17%)	1.1	2.09	0.04	1.0	1.1
Salary										
100–300	45	31	68.9	14	31.1	2.2	1.67	0.095	0.9	5.6
301–500	204	139	68.1	65	31.9	1.7	0.99	0.32	0.6	5.1
> 500	132	102	77.3	30	22.7	0.4	−1.56	0.12	0.1	1.3
No salary	219	150	68.5	69	31.5	Base category	-	-	-	-
Education										
No formal education	41	27	65.9	14	34.2	Base category	-	-	-	-
Primary education	80	55	68.8	25	31.3	1.0	−0.08	0.94	0.4	2.5
Secondary education	311	213	68.5	98	31.5	1.8	1.37	0.17	0.8	4.1
Tertiary education	168	127	75.6	41	24.4	2.5	1.87	0.06	1.0	6.5
Employment										
Student	53	39	73.6	14	26.4	Base category	-	-	-	-
Employed	251	174	69.3	77	30.7	11.4	2.37	0.02	1.5	85.9
Unemployed	143	101	70.6	42	29.4	23.3	3.03	0.00	3.0	179.2
Retired	29	18	62.1	11	37.9	50.4	3.52	0.00	5.7	449.5
House wife or husband	25	17	68.0	8	32.0	72.5	3.91	0.00	8.5	622.8
Self-employed	99	73	73.7	26	26.3	26.4	3.15	0.00	3.4	202.2

Note: For categorical variables, factors are shown as number (percentage) and medians [interquartile ranges (IQRs)] for continuous variables.

cons, Z – 3.88; 95% confidence interval – 0.0; 0.01. For categorical variables, factors are shown as number (percentage) and medians (interquartile ranges [IQR]) for continuous variables especially on the duration on HAART.

OR, odd ratio; BMI, body mass index; HAART, highly active antiretroviral therapy; HPT, hypertension.

### Prevalence of hypertension

The overall prevalence of hypertension in the study population was 29.7%. There was a higher prevalence of hypertension in men (31.6%) than women (28.1%), but this was not statistically significant (Table 2). Advanced age was significantly associated with a risk of hypertension. The prevalence of hypertension in participants aged 40 years and older was 29.9%, compared to 29.3% in participants younger than 40 years (*p* < 0.01).

### Social factors

Social factors such as area of residence and level of personal income showed no association with hypertension (Table 2). A positive or unknown family history of hypertension was positively correlated with the risk of hypertension (OR 2.00; 95% confidence interval [CI]: 1.00–4.1, *p* = 0.05 and OR 3.00; 95% CI: 1.6–5.5, *p* = 0.00, respectively)

### Overweight and obesity

A high prevalence of being overweight and obesity was noted, with 68.8% of the study participants found to have a BMI greater than 25 kg/m^2^. A greater number of participants in the hypertensive group were overweight or obese (70.8%) compared to the non-hypertensive group (68%). The mean BMI of participants in the hypertensive group was 29.6 kg/m^2^ compared to 29.2 kg/m^2^ in the normotensive group. There was a significant association between being overweight or obese and hypertension (OR: 0.50; 95% CI: 0.20–1.70) and (OR: 0.90; 95% CI: 0.30–2.90), respectively.

### Behavioural risk factors

Very few participants were reported to be smokers (*n* = 14). However, smokers were more likely to be hypertensive than non-smokers. A significant association between hypertension and smoking was observed (OR: 1.00; 95% CI: 0.231*–*3.792).

Only seven participants admitted to regular consumption of alcohol (beer) and a further 76 to occasional consumption. As reflected in Table 2, alcohol consumption increased the risk for the development of hypertension. The majority of participants (88.2%) engaged in daily physical activity, mainly occupational, household chores and travel related. Only a small proportion (11.8%) of participants did not engage in any regular physical activity. There was no association found between a sedentary lifestyle and hypertension.

### Highly active antiretroviral therapy, age and hypertension

As shown in Table 3, in order to test the association between age and duration on HAART in influencing the likelihood of a patient to be hypertensive, a multivariate logistic regression is undertaken with previously used age and HAART categories as well as BMI and gender as independent variables. Compared to those patients with ≤ 40 years and being on HAART less than 5 years, the odds of being hypertensive significantly increased in older ages and with longer duration on ART. Participants aged 60 years and older and those who had been on ART between 5 and 10 years had the highest odds ratio (OR: 8.77; 95% CI: 3.28–23.47). All HAART categories associated with age ≤ 40 years, gender and BMI categories had statistically insignificant ORs (*p* > 0.01).

**TABLE 3:** Multivariate logistic regression analysis of factors associated with hypertension in human immunodeficiency virus–positive patients.

Variable	Odds ratio	s.e.	*z*	*P* > |*z*|	95% confidence interval	
					From	To
Age						
≤ 40, HAART ≤ 5	Base category	-	-	-	-	-
≤ 40, HAART > 5 & ≤ 10	1.38	0.47	0.96	0.34	0.71	2.69
≤ 40, HAART > 10	0.58	0.34	−0.93	0.35	0.18	1.85
40–59, HAART ≤5	0.87	0.33	−0.38	0.71	0.41	1.84
40–59, HAART > 5 & ≤ 10	3.27	1.05	3.71	0.00	1.75	6.13
40–59, HAART > 10	2.41	0.96	2.21	0.03	1.10	5.26
≥ 60, HAART ≤ 5	2.16	1.38	1.21	0.02	0.62	7.54
≥ 60, HAART > 5 & ≤ 10	8.77	4.40	4.32	0.00	3.28	23.47
≥ 60, HAART > 10	5.64	3.28	2.98	0.00	1.80	17.64
Gender						
Female	Base category	-	-	-	-	-
Male	0.90	0.17	−0.54	0.59	0.62	1.32
BMI						
Below 18.5	Base category	-	-	-	-	-
18.5–24.9	0.88	0.46	−0.24	0.81	0.32	2.44
25.0–29.9	0.89	0.46	−0.23	0.82	0.32	2.45
> 30.0	1.77	0.89	1.14	0.26	0.66	4.73
Constant	0.20	0.11	−2.88	0.00	0.07	0.60

BMI, body mass index; HAART, highly active antiretroviral therapy; s.e., standard error.

## Discussion

The hypertension prevalence rate of 29.7% reported in this study among HIV-positive people on HAART is similar to that estimated in other HIV-positive populations, and in the general population of sSA.^11,18^ However, the limited data from sSA countries require larger studies, and studies comparing HIV-negative to HIV-positive populations. Previous studies by Pefura Yone et al.^19^ Ekali et al.,^20^ Muhammad et al.^21^ and Njelekela et al.^22^ revealed a higher prevalence of hypertension in HIV-positive patients on HAART compared to HIV-negative people. This is contrary to findings of other studies like that of Dimala et al.^23^ who show that a lower prevalence of hypertension was noted in HIV-positive people receiving HAART. The prevalence rate recorded in this study is comparable to the prevalence rate reported by Mutede et al.^24^

An age of older than 40 years, smoking, alcohol consumption, a family history of hypertension and overweight were all associated with a risk of hypertension. These associations have been previously established in sSA populations, but not in the HIV-positive sub-population.^25^

Gender was not a risk factor for hypertension. Advanced age was significantly associated with a risk of hypertension. In both genders, those who were 40 years and older had a statistically greater risk of being hypertensive than those younger than 40 years. However, the prevalence of hypertension in people younger than 40 years was notably high (29.3%). This suggests that people under 40 years who are on HAART are more likely to develop hypertension than their HIV-negative peers. Further research comparing HIV prevalence in HIV-positive and negative people under 40 years is needed. A high prevalence of hypertension in young adults would potentially result in premature cardiovascular morbidities and death.

Patients on HAART in this clinic, as elsewhere in sSA, had a high prevalence of being overweight and obesity.^26,27^ A BMI greater than 25 kg/m^2^ increased the odds of being hypertensive.^28^ A greater proportion of women than men with hypertension were found to be overweight or obese. Being overweight, but not obese, was associated with an increased risk of hypertension. A larger sample size would perhaps yield a significant positive association of obesity with hypertension.

Social factors such as marital status, income and area of residence showed no association with hypertension. However, the populations sampled in this study were mostly urban based, with high levels of literacy and personal income. These results cannot be extrapolated to more rural communities.

Despite low reported rates of smoking and alcohol consumption, both of these factors were significantly associated with hypertension. Although a lack of regular physical activity was not an independent risk factor for hypertension in this study, exercise should still be encouraged among all HIV-positive people on HAART for its global health benefits, especially in case of other cardiovascular risk factors such as high BMI and hypertension.

Higher mean values of age and duration of HIV infection were observed. This could probably be attributed to the increased life expectancy of HIV-positive people since the introduction of HAART. The risk of developing hypertension increased with age and the duration on HAART. Participants who were aged 60 years and above and had been on HAART for 5 years and above had a significantly increased risk of hypertension.

Screening for hypertension needs to be improved as demonstrated in this study. Despite regular clinic attendance, there were still participants enrolled at the clinic who were found to have undiagnosed hypertension. In the hypertensive group, 11.2% (*n* = 20) of participants were not previously diagnosed. Adherence levels to anti-hypertensive medication were only 62%. This highlights the need to strengthen health systems in order to improve screening, diagnosis and monitoring for chronic NCDs. Integration of services can also help address co-morbidities in people with chronic diseases.

In this study of HIV-positive people on HAART, the prevalence of hypertension was high. Possible factors for the high prevalence rate of hypertension in HIV-positive populations in sSA include differences in HIV disease progression, diet, HAART regimens and health infrastructure.^29^

### Limitations

Our primary analyses were based on a descriptive, cross-sectional study; hence, transience between causes and development of hypertension could not be established. Data were collected over a 3-month period and may not necessarily reflect the annual patient profile. The study did not include an HIV-negative control group for comparison. Data regarding adherence and socio-economic background required participants’ disclosure of this information. The reliability of this data would depend on participants’ truthfulness. Validity of data from participants is limited by the fact that it rarely can be independently verified, and this leaves room for information bias. The study was conducted in an urban-based clinic in Zimbabwe, and the findings may not be extrapolated to rural populations.

Prolonged exposure to HAART is associated with older age which is a confounding factor in the evaluation of the duration of HAART as an independent risk factor.

## Conclusion

Hypertension is as common among HIV-positive people as in HIV-negative people. Risk factors for hypertension in this HIV-positive population were: age over 40 years, obesity and being overweight, smoking and alcohol consumption and a family history of hypertension. Research findings show no difference between protease-inhibitor-based and other regimens with a risk of hypertension. This suggests that exposure to protease inhibitors is not a risk factor for hypertension in this HIV-positive population. Young adults were found to be developing hypertension despite having few co-morbidities. Further studies are needed to understand which risk factors in HIV-positive people predispose them to hypertension and at an earlier age.

The need for an integrated approach to primary care is advocated. An integrated chronic care model will improve detection and treatment of co-morbidities in the HIV population in sSA. Greater vigilance is also required by health care providers to screen and treat HIV-positive patients for hypertension, particularly in participants older than 40 years, and participants who have been on HAART for more than 10 years. The number of older adults on HAART will increase because of the increased life expectancy of HIV-positive individuals. Health providers working with this population need to be cognizant of this.^30^ The researchers recommend the use of the Innovative Care for Chronic Conditions Framework to provide guidance in restructuring vertical programmes such as the HIV/ART programme to be a more patient-centred model.^30,31,32^

## Footnotes

1 The word STEP in the approach denotes the three steps taken in the surveillance approach. Step 1: Is surveillance of social demographic/key behavoural variables; Step 2: Is physical measurements and Step 3: Is biochemical measurements.
